# Association between changes in sleep duration and the risk of incident depressive symptoms among Chinese middle-aged and older adults

**DOI:** 10.1371/journal.pone.0329797

**Published:** 2025-08-11

**Authors:** LiHan Lin, YiLing Dai, JiaJun Huang, Wei Zheng, YiPing Liu

**Affiliations:** 1 College of Physical Education, Huaqiao University, Quanzhou, China; 2 Provincial University Key Laboratory of Exercise and Health Science, School of Physical Education and Sport Science, Fujian Normal University, Fuzhou, China; 3 School of Medicine, Xiamen University, Xiamen, China; 4 School of Physical Education and Health Care, Sanming University, Sanming, China; Harbin Institute of Technology, CHINA

## Abstract

**Background:**

Although insufficient sleep is a known risk factor for depressive symptoms among middle-aged and older adults, prior studies have mostly focused on baseline sleep duration. This study investigates the association between changes in sleep duration and the risk of incident depressive symptoms in this population.

**Methods:**

This study utilized data from the China Health and Retirement Longitudinal Survey (CHARLS), covering waves from 2011 to 2018. Sleep duration was assessed through self-report and categorized as short (<6 h/day), normal (6–8 h/day), and long (> 8 h/day). Changes in sleep duration were determined based on measurements at baseline and the 2-year follow-up. Depressive symptoms were evaluated using the 10-item Center for Epidemiological Studies Depression Scale (CESD-10). Hazard ratios (HRs) and 95% confidence intervals (CIs) were estimated using Cox proportional hazards models after adjusting for potential confounders, and sensitivity analyses were conducted to assess the robustness of the associations by applying alternative cut-off values for sleep duration classification and by conducting stratified subgroup analyses.

**Results:**

A total of 5,636 participants (female: 44.7%, mean age: 58.1 ± 8.8 years) were included. During a median follow-up of 4.14 years, 1,656 participants developed depressive symptoms. Short sleep at baseline was associated with a higher risk of depressive symptoms (HR = 1.33, 95% CI: 1.22–1.45). A change from normal to short sleep further increased the risk (HR = 1.48, 95% CI: 1.24–1.76), suggesting a greater hazard than baseline short sleep alone. A change from long to short sleep also elevated the risk (HR = 1.49, 95% CI: 1.16–1.91). These associations remained robust in sensitivity analyses.

**Conclusion:**

Among Chinese adults aged 45 years and older, maintaining a normal sleep duration and avoiding a shift to short sleep may help prevent the onset of depression. Individuals who experience a marked decline in sleep duration should be considered a key target population for the prevention of depressive symptoms.

## Introduction

Depression is a common mental health issue and has become one of the leading psychiatric disorders globally [1]. Recent estimates from the World Health Organization indicate that depression affects over 280 million individuals globally [2]. In the context of rapid societal changes, China has witnessed an increase in the age-standardized prevalence of depression, rising from 3,224.6 per 100,000 in 1990–3,990.5 per 100,000 in 2017, with disproportionately higher rates observed among older adults [3,4]. Depression not only exerts a profound negative impact on quality of life but is also associated with an elevated risk of cognitive decline, functional impairment, cardiovascular disease, type 2 diabetes, and premature mortality [5–8]. Accordingly, identifying and implementing early intervention in high-risk populations is of critical public health importance.

With advancing age, individuals commonly experience changes in sleep patterns, including decreased total sleep duration, increased sleep fragmentation, and frequent awakenings [9,10]. These age-related sleep disturbances, particularly short sleep duration, have been associated with an increased risk of depressive symptoms in middle-aged and older adults [11–14]. However, these studies primarily focused on cross-sectional assessments of baseline sleep duration, without considering changes in sleep duration status over time. For instance, Luo et al. found that short sleep duration was associated with an increased risk of depressive symptoms in a large cross-sectional sample of Chinese adults [12]. Baseline sleep assessments reflect only a single time point and may not account for within-person fluctuations in sleep patterns that are common among middle-aged and older adults. These fluctuations often reflect age-related physiological changes, such as reduced sleep efficiency and shortened total sleep duration, which may evolve over time and influence mental health outcomes. Compared to a single baseline assessment, studying changes in sleep duration during follow-up may better capture the dynamic biological associations, such as the relationship between changes in sleep duration, either increasing or decreasing, and the incidence of depressive symptoms. Basic research suggests that the physiological mechanisms linking short sleep duration to the onset of depressive symptoms may involve common biological pathways, such as increased inflammation, metabolic dysregulation, and neurotransmitter imbalance [15,16]. More importantly, evidence indicates that normal sleep duration can be maintained through interventions such as cognitive behavioral therapy, pharmacological treatment, lifestyle changes, exercise, and sleep hygiene [17–20]. Therefore, assessing the risk of depressive symptoms in individuals who experience a change from normal to short sleep duration will provide crucial evidence for early intervention and timely identification of individuals at risk.

In this study, we utilized data from the China Health and Retirement Longitudinal Study (CHARLS) to investigate the association between changes in sleep duration and the risk of incident depressive symptoms. We hypothesize that progression from normal or long sleep to short sleep increases the risk of developing depressive symptoms, whereas recovery from short to normal or long sleep may reduce this risk.

## Materials and methods

### Data source

The China Health and Retirement Longitudinal Study (CHARLS) is a nationally representative and prospective cohort conducted in China. The survey covers 150 counties and 450 communities (villages) across 28 provinces, gathering longitudinal data from a nationally representative sample of individuals over 45 years old through face-to-face household interviews. This data encompasses various dimensions, including socioeconomic status and health conditions, providing a robust foundation for research in aging science. The ethics approval for the collection of CHARLS data has been approved by the Peking University Biomedical Ethics Review Committee (IRB00001052–11015). Written informed consent was obtained from all participants or their legal agents before the commencement of any study process. Detailed descriptions, including the sampling procedures, questionnaire, and the raw data used in this study can be accessed at https://charls.pku.edu.cn and the supplementary materials (S1 File, and S2 Table).

### Study population

**Fig 1:**
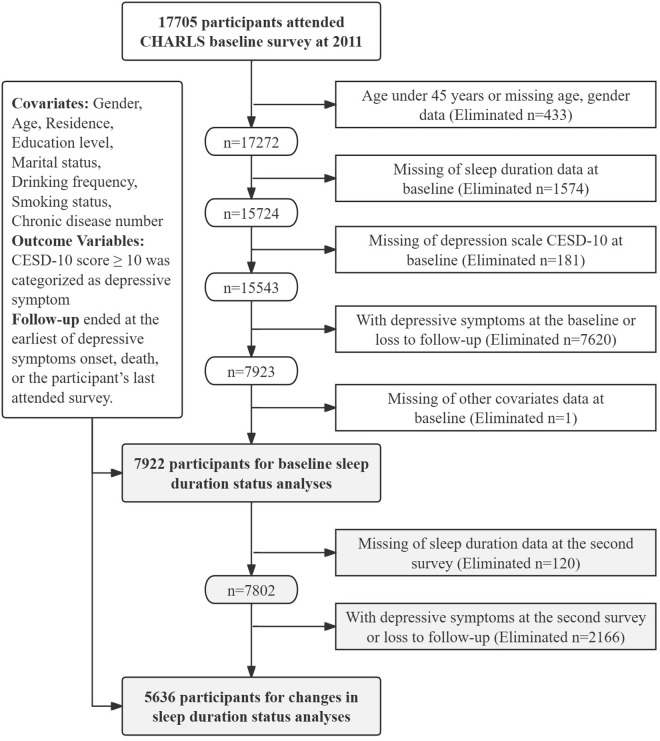
Selection process for the study population. A total of 17,705 participants were recruited in the 2011 CHARLS baseline survey. The upper part shows the sample selection for the analysis of baseline sleep duration status (n = 7,922). The lower part shows the subsample (n = 5,636) used for the analysis of changes in sleep duration status among those with complete follow-up and no depression at the second survey.

This study utilized data from the CHARLS collected between 2011 and 2018, as the 2020 wave was affected by COVID-19, resulting in fewer survey items and lower response rates, as noted in the S1 File. **Methods supplement**. In this study, wave 1 (2011) was considered the baseline, and wave 2 (2013) was the second survey. Participants were followed for outcomes through wave 4 (2018). Fig 1 shows the selection process of the CHARLS study population. Among 17,705 participants from CHARLS, we applied the following exclusion criteria to define the analytical sample: (1) individuals younger than 45 years or with missing age or gender information; (2) participants with missing baseline sleep duration data; (3) those with missing baseline CESD-10 depression scale data. (4) those with depressive symptoms at baseline or who were lost to follow-up; and (5) those with missing data on other covariates at baseline. After applying these criteria, a total of 7,922 eligible participants were included in baseline sleep duration analyses. For changes in sleep duration status analyses, we further excluded 2,559 participants according to similar criteria. The remaining 5,636 participants were included in the final analysis.

### Assessment of sleep duration

**Fig 2:**
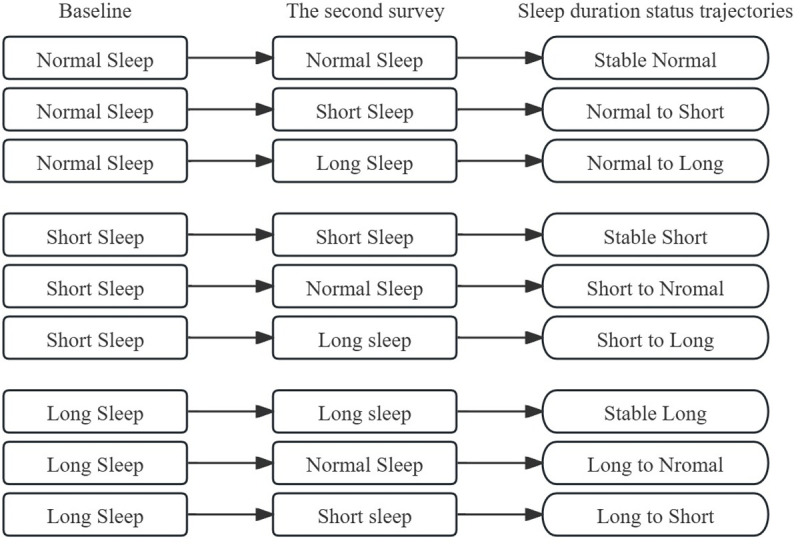
Trajectory of Changes in Sleep Duration Status. Sleep duration trajectories were defined by combining sleep duration status at baseline and the second survey, resulting in nine distinct patterns.

The assessment relied on self-reported sleep duration, a widely used measure in sleep research [21–24]. Sleep duration was assessed using the self-reported question: “During the past month, how many hours of actual sleep did you get at night (average hours for one night)?” Based on national recommendations and previous studies on sleep and depressive symptoms among Chinese middle-aged and older adults [11,25,26], sleep duration was classified into three statuses: short sleep (< 6 h/day), normal sleep (6–8 h/day), or long sleep (> 8 h/day). Sleep duration status was assessed at baseline (2011, Wave 1) and the 2-year follow-up (2013, Wave 2). Based on changes between the two waves, participants were classified into nine sleep duration trajectory patterns, as shown in Fig 2. Objective measures of sleep duration, such as actigraphy or polysomnography, were not feasible in this large-scale, nationally representative study, which included over 20,000 participants in China, due to logistical complexity and high costs. Nonetheless, validation studies in similar populations have shown a moderate correlation with objective measures such as actigraphy, supporting the validity of our data [27,28].

### Depressive symptoms ascertainment and follow-up

Depressive symptoms were assessed using the 10-item Center for Epidemiologic Studies Depression Scale (CESD-10), a validated instrument widely used in China to evaluate mood and behavioral symptoms over the past week [29,30]. Each item was rated on a 4-point Likert scale ranging from “rarely or none of the time (<1 day)” to “most or all of the time (5–7 days)”. For the 8 negatively worded items, responses were scored from 0 to 3, whereas for the 2 positively worded items, scores were reverse-coded. The total score ranged from 0 to 30, with a score ≥10 indicating clinically significant depressive symptoms.

In the baseline sleep duration status analyses, follow-up was initiated from wave 1 (2011) of CHARLS. For sleep duration status change analyses, follow-up began at wave 2 (2013). The endpoint of follow-up was defined as the first occurrence of depressive symptoms, death, or the censoring date, whichever came first. The censoring date was the date of the last survey attended by each participant, ideally wave 4 (2018).

### Covariates

Based on previous studies [31–33], eight covariates associated with the onset of depressive symptoms were included in the analysis, including gender (male, female), age (45–54, 55–64, 65–74, ≥ 75 years), education level (≤primary school, middle school, ≥ high school), marital status (married/cohabiting, widowed, divorced/never married/other), residence (rural or urban), drinking frequency (>once/month, ≤ 1/month, never), smoking status (yes, no) and the chronic disease number. The number of chronic diseases was derived from self-reported physician diagnoses, based on whether a doctor had ever diagnosed the participant with any of the following 12 conditions: hypertension, dyslipidemia, diabetes, cancer, chronic lung disease, liver disease, heart disease, stroke, kidney disease, digestive disease, arthritis, and asthma. Participants were categorized into three groups according to the number of reported conditions: 0 (none), 1 (single condition), and ≥2 (multimorbidity). Other potential confounders, including baseline physical activity, cognitive function, social engagement, and anxiety symptoms, were considered during model development but were not included in the final models (see S1 File. **Methods supplement** for details).

### Statistical analysis

For descriptive statistics, participant characteristics were presented as frequencies (n) and percentages (%). To examine the association between baseline sleep duration status and the risk of incident depressive symptoms, Cox proportional hazards regression was used to estimate hazard ratios (HRs) and 95% confidence intervals (CIs). Two models were fitted using participants with normal sleep duration as the reference group. Model 1 was unadjusted. Model 2 was adjusted for gender, age, education level, marital status, residence, drinking frequency, smoking status, and number of chronic diseases. We further analyzed the associations between changes in sleep duration status and the risk of incident depressive symptoms using similar methods. The proportional hazards assumption was assessed using Schoenfeld residuals and found to be satisfied for all Cox models [34].

Three sensitivity analyses were conducted: (i) Baseline characteristics of included participants were compared with those of the full baseline cohort to evaluate potential selection bias. (ii) Because the cut-off values for defining sleep duration status remain debated, we additionally applied an alternative classification proposed in a Joint Consensus Statement issued by the American Academy of Sleep Medicine and the Sleep Research Society [35], in which sleep duration was categorized as short sleep (<7 h/day), normal sleep (7–9 h/day), and long sleep (>9 h/day). (iii) To evaluate the robustness of our findings, we stratified the Cox regression models by key subgroups, including gender, age group, and number of chronic diseases. These subgroup analyses were conducted separately for both baseline sleep duration status and changes in sleep duration status. Interaction terms between sleep duration indicators and subgroup variables were also tested to assess potential effect modification. All statistical analyses were conducted using SPSS Statistics version 26.0. All P-values were two-sided, and P < 0.05 was considered statistically significant.

## Results

### Baseline characteristics of the study population

Based on the predefined inclusion and exclusion criteria, 7,922 participants (47.2% female; mean age = 58.5 ± 9.1 years) were included in the baseline sleep duration status analysis. During a median follow-up of 5.15 years, 3,240 participants developed depressive symptoms. Compared with adults who reported the recommended 6–8 h of sleep, short sleepers (< 6 h) were older, more often widowed, rural residents with lower educational attainment and a higher prevalence of multimorbidity (≥ 2 chronic diseases). Long sleepers (> 8 h) shared the disadvantage of lower education and predominantly rural residence but, unlike short sleepers, had the largest proportion free of chronic conditions. Gender, smoking, and drinking patterns were broadly similar across the three sleep‑duration groups. The baseline characteristics of the study population are summarized in Table 1.

**Table 1:** Baseline characteristics of participants for baseline sleep duration status analyses, expressed as n (%).

Characteristics	Sleep duration status		
	Short, < 6 h/day (n = 1634)	Normal, 6–8 h/day (n = 3590)	Long, > 8 h/day (n = 2699)
**Gender**			
Male	821 (50.2)	1928 (53.7)	1434 (53.1)
Female	813 (49.8)	1661 (46.3)	1265 (46.9)
**Age (years)**			
45-54	473 (28.9)	1424 (39.7)	1041 (38.6)
55-64	661 (40.5)	1387 (38.6)	998 (37.0)
65-74	363 (22.2)	604 (16.8)	492 (18.2)
≥75	137 (8.4)	174 (4.8)	168 (6.2)
**Education level**			
≤primary school	1131 (69.2)	2029 (56.5)	1708 (63.3)
Middle school	334 (20.4)	915 (25.5)	636 (23.6)
≥high school	169 (10.3)	645 (18.0)	355 (13.2)
**Marital status**			
Married/partnered	1433 (87.7)	3316 (92.4)	2461 (91.2)
Widowed	173 (10.6)	220 (6.1)	197 (7.3)
Divorced/never married/others	28 (1.7)	53 (1.5)	41 (1.5)
**Residence**			
Rural	968 (59.2)	1962 (54.7)	1671 (61.9)
Urban	666 (40.8)	1627 (45.3)	1028 (38.1)
**Drinking frequency**			
>1/month	472 (28.9)	1032 (28.8)	718 (26.6)
≤1/month	110 (6.7)	332 (9.3)	212 (7.9)
Never drank	1052 (64.4)	2225 (62.0)	1769 (65.5)
**Smoke**			
Yes	670 (41.0)	1513 (42.2)	1107 (41.0)
No	964 (59.0)	2076 (57.8)	1592 (59.0)
**Chronic disease number**			
0	543 (33.2)	1396 (38.9)	1124 (41.6)
1	494 (30.2)	1120 (31.2)	841 (31.2)
≥2	597 (36.5)	1073 (29.9)	734 (27.2)

Baseline sleep duration and covariates were obtained from wave 1 (2011) of the CHARLS survey.

For the analysis of changes in sleep duration status, 5,636 participants (44.7% female; mean age = 58.1 ± 8.8 years) were included according to the corresponding criteria. During a median follow-up of 4.14 years, 1,656 participants developed depressive symptoms. The baseline characteristics of these participants are presented in Table 2.

**Table 2:** Baseline characteristics of participants for changes in sleep duration status analysis, expressed as n (%).

Characteristics	Sleep duration status		
	Short, < 6 h/day (n = 1075)	Normal, 6–8 h/day (n = 2583)	Long, > 8 h/day (n = 1978)
**Gender**			
Male	577 (53.7)	1451 (56.2)	1089 (55.1)
Female	498 (46.3)	1132 (43.8)	889 (44.9)
**Age (years)**			
45-54	323 (30.0)	1026 (39.7)	783 (39.6)
55-64	446 (41.5)	1022 (39.6)	749 (37.9)
65-74	233 (21.7)	428 (16.6)	348 (17.6)
≥75	73 (6.8)	107 (4.1)	98 (5.0)
**Education level**			
≤primary school	734 (68.3)	1421 (55.0)	1218 (61.6)
Middle school	230 (21.4)	687 (26.6)	506 (25.6)
≥high school	111 (10.3)	475 (18.4)	254 (12.8)
**Marital status**			
Married/partnered	968 (90.0)	2402 (93.0)	1819 (92.0)
Widowed	89 (8.3)	144 (5.6)	132 (6.7)
Divorced/never married/others	18 (1.7)	37 (1.4)	27 (1.4)
**Residence**			
Rural	636 (59.2)	1437 (55.6)	1223 (61.8)
Urban	439 (40.8)	1146 (44.4)	755 (38.2)
**Drinking frequency**			
>1/month	331 (30.8)	790 (30.6)	552 (27.9)
≤1/month	72 (6.7)	243 (9.4)	161 (8.1)
Never drank	672 (62.5)	1550 (60.0)	1265 (64.0)
**Smoking status**			
Yes	459 (42.7)	1129 (43.7)	831 (42.0)
No	616 (57.3)	1454 (56.3)	1147 (58.0)
**Chronic disease number**			
0	370 (34.4)	1056 (40.9)	863 (43.6)
1	340 (31.6)	799 (30.9)	594 (30.0)
≥2	365 (34.0)	728 (28.2)	521 (26.3)

Baseline sleep duration and covariates were obtained from wave 1 (2011) of the CHARLS survey.

### Association of baseline sleep duration status with incident depressive symptoms

Table 3 summarizes the associations between baseline sleep‑duration status and the risk of depressive symptoms. After adjusting for confounders, participants with short sleep (<6 h/day) had a significantly higher risk of developing depressive symptoms compared to those with normal sleep (6–8 h/day) (HR 1.33, 95% CI 1.22–1.45, *P* < 0.001). In contrast, long sleep (>8 h/day) was not associated with an altered risk (HR 0.98, 95% CI 0.90–1.06).

**Table 3:** Association of baseline sleep duration status with risk of incident depression symptoms.

	No. of cases/total	Model 1		Model 2	
		HR (95%CI)	P	HR (95%CI)	P
Normal (6–8 h/day)	1382/3589	1(reference)		1(reference)	
Short sleep (<6 h/day)	812/1634	1.43 (1.31-1.56)	**<0.001**	1.33 (1.22−1.45)	**<0.001**
Long sleep (>8 h/day)	1046/2699	1.00 (0.93-1.09)	0.908	0.98 (0.90-1.06)	0.598

CI = Confidence Interval; HR = Hazard Ratio.

Model 1, without adjustment; Model 2 was adjusted for gender, age, education level, marital status, residence, drinking frequency, smoking status, and number of chronic diseases.

### Association of changes in sleep duration status with incident depressive symptoms

**Fig 3:**
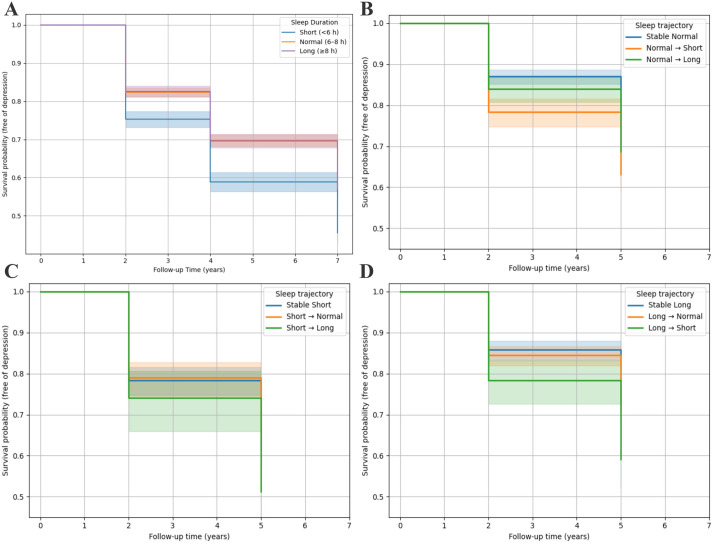
Kaplan–Meier curves for incident depressive symptoms by sleep duration status and changes. Participants with short sleep at baseline had the highest risk of developing depressive symptoms. (B) Individuals with baseline normal sleep who transitioned to short sleep had a higher risk of developing depressive symptoms. (C) Among baseline short sleepers, no substantial differences in depression-free survival were observed across different sleep trajectories. (D) Among participants with long sleep at baseline, transitioning to short sleep was linked to an increased risk of depressive symptoms.

Table 4 shows the number and percentage of changes in sleep duration status after two years of follow-up. Among participants with normal sleep at baseline, 551 (21.3%) transitioned to short sleep or 581 (22.5%) to long sleep. Meanwhile, among those with short sleep at baseline, 371 (34.5%) improved to normal sleep or 139 (12.9%) extended to long sleep. Of those with baseline long sleep, 889 (44.9%) transitioned to normal sleep, and 236 (11.9%) shortened to <6 h/day. Table 5 shows the association between changes in sleep duration status and the risk of incident depressive symptoms. Compared with participants who maintained stable normal sleep, those who transitioned from normal to short sleep had a significantly elevated risk of developing depressive symptoms (HR 1.48, 95% CI 1.24–1.76, *P* < 0.001). Similarly, among participants with baseline long sleep, those who shifted to short sleep also showed an increased risk (HR 1.49, 95% CI 1.16–1.91, *P* = 0.002). However, a change from short to normal sleep duration did not significantly reduce the risk of developing depressive symptoms (HR 0.90, 95% CI 0.72–1.13, *P* = 0.359). Kaplan–Meier analysis showed patterns consistent with the Cox regression results, with short sleep-in baseline and transitions to short sleep associated with a lower probability of remaining free of depressive symptoms (Fig 3).

**Table 4:** Number and percentage of the changes in sleep duration status.

Baseline sleep duration status	The second survey sleep duration status	CHARLS 2011–2018	
n	%
Normal sleep (6–8 h/day)	Normal sleep	1451	56.17
	Short sleep	551	21.33
	Long sleep	581	22.49
Short sleep (<6 h/day)	Normal sleep	371	34.51
	Short sleep	565	52.56
	Long sleep	139	12.93
Long sleep (>8 h/day)	Normal sleep	889	44.94
	Short sleep	236	11.93
	Long sleep	853	43.12

The time interval between baseline and the second survey was two years in the CHARLS (2011–2013).

**Table 5:** Association of changes in sleep duration status with risk of incident depression symptoms.

	No. of cases/total	Model 1		Model 2	
		HR (95%CI)	P	HR (95%CI)	P
Stable Normal	350/1451	1(reference)		1(reference)	
Normal to Short	190/551	1.57 (1.32-1.88)	**<0.001**	1.48 (1.24-1.76)	**<0.001**
Normal to Long	169/581	1.25 (1.04-1.51)	**0.016**	1.12 (0.93-1.35)	0.221
Stable Short	204/565	1(reference)		1(reference)	
Short to Normal	127/371	0.92 (0.74-1.15)	0.472	0.90 (0.72-1.13)	0.359
Short to Long	60/139	1.33 (0.99-1.77)	0.055	1.26 (0.94-1.68)	0.124
Stable Long	224/853	1(reference)		1(reference)	
Long to Normal	244/889	1.03 (0.86-1.23)	0.781	1.05 (0.88-1.26)	0.592
Long to Short	88/236	1.55 (1.21-1.99)	**<0.001**	1.49 (1.16-1.91)	**0.002**

CI = Confidence Interval; HR = Hazard Ratio.

Model 1, without adjustment; Model 2 was adjusted for gender, age, education level, marital status, residence, drinking frequency, smoking status, and number of chronic diseases.

### Sensitivity analyses

Sensitivity analyses showed that the analytic sample was generally comparable to the full baseline cohort for most characteristics. Significant differences were observed for age, education level, and residence, mainly reflecting greater loss to follow-up among older and less-educated participants (Supplementary Table, Table S1 in S1 Table). The associations of baseline sleep duration status and changes in sleep duration status with incident depressive symptoms remained generally consistent when applying alternative cut-off values (Supplementary Table, Table S2–S3 in S1 Table). In the adjusted model, short sleep was still significantly associated with a higher risk of depressive symptoms compared to normal sleep in the baseline analysis (HR = 1.26, 95% CI: 1.17–1.35, *P* < 0.001). In the trajectory analysis, the association between transition from normal to short sleep and increased depression risk remained significant (HR = 1.29, 95% CI: 1.12–1.48, *P* < 0.001), while the previously significant association for the “Long to Short” group became non-significant (HR = 1.51, 95% CI: 0.75–3.03, **P* *= 0.243), likely due to the small sample size (23/53).

The associations of baseline and changes in sleep duration status with incident depressive symptoms were generally consistent across subgroups (Age, gender, and number of chronic diseases). For baseline sleep duration status, subgroup analyses were conducted comparing short sleepers with normal sleepers (Supplementary Table, Table S4 in S1 Table); for changes in sleep duration, subgroup analyses focused on Normal-to-Short versus Stable Normal and Long-to-Short versus Stable Long patterns (Supplementary Table, Tables S5–S6 in S1 Table), which were previously identified as significantly associated with incident depressive symptoms. All subgroup effects were not significantly modified (P for interaction > 0.05), which implies that the association between sleep duration and its changes and depressive symptoms is robust across all subgroups.

## Discussion

In this nationwide prospective cohort study of Chinese adults aged 45 years and older, we examined the associations of baseline sleep duration and its changes with the risk of incident depressive symptoms. Compared to individuals with normal sleep duration, those with short sleep had a significantly higher risk of developing depressive symptoms. Moreover, participants who changed from normal to short sleep exhibited a higher risk than those who maintained normal sleep duration, and this association remained robust across alternative cut-off definitions and subgroup analyses. Additionally, individuals who changed from long to short sleep also showed an elevated risk of depressive symptoms compared to those who maintained a long sleep duration.

Previous studies have shown a close association between sleep duration and mental disorders, particularly depressive symptoms. Previous studies on Chinese populations have shown a close association between sleep duration and mental disorders, particularly depressive symptoms. A study from the China Kadoorie Biobank, which involved over 500,000 participants, found that short sleep duration (<6 hours) was significantly associated with a high risk of incident depression symptoms [36]. In another Chinese rural cohort study, short sleep duration (<6 hours) was also linked to elevated depressive symptoms [37]. Consistent results were also observed in our study: even after controlling traditional mental health risk factors, individuals with sleep duration of less than 6 hours had a significantly higher risk of depressive symptoms compared to those with normal sleep duration, further supporting the notion that insufficient sleep is an independent risk factor for depressive symptoms.

In addition to baseline sleep duration, our study also examined the association between changes in sleep duration and the incidence of new-onset depressive symptoms, which have not been previously discussed in the literature. We found that individuals who transition from stable normal sleep to short sleep had a significantly increased risk of developing depressive symptoms. This finding highlights the adverse impact of reduced sleep duration on the onset of depressive symptoms. Additionally, when individuals with long sleep durations transitioned to short sleep, the risk of new-onset depressive symptoms also increased. In contrast, individuals who recovered from short sleep to normal sleep exhibited a lower risk of developing depressive symptoms, although this difference did not reach statistical significance. When using an alternative set of sleep duration cut-offs (<7, 7–9, > 9 hours) to define sleep status and conducting subgroup analyses based on age, gender, and the number of chronic diseases, the results remained generally consistent, further corroborating our previous findings. The increased risk of depressive symptoms observed among individuals transitioning from normal or long to short sleep duration may be explained by several underlying neurobiological and physiological mechanisms. Abrupt reductions in sleep duration can disrupt circadian rhythms and the homeostatic regulation of sleep [38,39], leading to dysregulation of the hypothalamic–pituitary–adrenal axis and increased cortisol secretion, both of which are associated with the onset of depression [40,41]. Additionally, these changes may induce low-grade inflammation [42] and negatively affect neural circuits involved in mood regulation, such as the prefrontal cortex and hippocampus [43].

Given these risks, strategies to prevent or reduce transitions from normal or long to short sleep duration are needed. Accordingly, our findings have important clinical and public health implications. First, the assessment of sleep duration changes should be integrated into routine mental health or psychiatric evaluations, particularly for individuals aged 45 and older. Individuals with short sleep duration should be considered a primary target for preventing depressive symptoms. Regular sleep assessments should also be conducted for individuals with normal sleep to identify those at high risk of depression due to shortened sleep duration, allowing for early intervention. Several intervention measures—such as promoting good sleep hygiene [44], cognitive behavioral therapy for insomnia (CBT-I) [45], regular physical activity [46], and the management of chronic medical conditions [47]—have been shown to help maintain healthy sleep patterns and may prevent the transition from normal or long to short sleep duration. In conclusion, effective interventions are needed to help maintain healthy sleep patterns in middle-aged and older adults to prevent the onset of depressive symptoms.

This study has several limitations. First, consistent with previous research [48–50], sleep duration was determined based on self-reported data, which may be subject to measurement error and misclassification of sleep status. Second, due to the impact of the COVID-19 pandemic on the CHARLS 2020 wave, changes in sleep duration were assessed using only two survey waves, with the overall follow-up conducted between 2011 and 2018. Third, due to the observational design of the study, causal inferences cannot be made regarding the relationship between changes in sleep duration and the incidence of depressive symptoms.

## Conclusions

Different changes in sleep duration status are associated with varying risks of incident depressive symptoms. Changes from long or normal sleep duration to short sleep duration are linked to an increased risk of depressive symptoms. Future research is warranted to develop targeted prevention strategies aimed at mitigating the adverse effects of sleep reduction and to design effective interventions for maintaining healthy sleep duration among middle-aged and older adults.

## Supporting information

S1 File Methods supplement. (PDF)

S1 Table Supplementary Table S1-S6. (XLSX)

S2 Table Raw data. (XLSX)
