# Supplementary material for: Association between changes in sleep duration and the risk of incident depressive symptoms among Chinese middle-aged and older adults
Source: PLoS One. 2025 Aug 11;20(8):e0329797. doi: 10.1371/journal.pone.0329797 (PMC12338831; doi:10.1371/journal.pone.0329797)
Supplement: S1 File — (PDF) [file pone.0329797.s001.pdf]

S3 Methods supplement

Rationale for Excluding the 2020 CHARLS Wave

The 2020 wave (Wave 5) of the China Health and Retirement Longitudinal Study (CHARLS) was not included in our analysis due to substantial methodological and content-related disruptions resulting from the COVID-19 pandemic. As documented in the Wave 5 User Guide (https://charls.charlsdata.com/pages/Data/2020-charls-wave5/en.html), CHARLS explicitly acknowledged that the pandemic had a profound impact on survey implementation.

First, as noted in Chapter 1 (General Introduction):"In late 2019 and early 2020, there was an outbreak of Covid-19 in China. In order to document the impact of the pandemic on the lives and health of middle-aged and elderly people in China, the information related to Covid-19 was additionally collected in wave 5."

This led to the inclusion of a dedicated COVID-19 module, which extended interview time and consequently reduced the breadth of information collected in other areas. As clarified in Chapter 3 (Survey Content):

"What needs to be pointed out is that, due to the extra interview time added by the new COVID module, less information was collected in some other modules in Wave 5 compared to previous waves. For example, information on siblings was not collected in the family module; additionally, there was less information on health status, health care utilization, health insurance, pension and assets in this survey."

Furthermore, the pandemic altered the data collection methods, introducing inconsistencies with previous waves. According to Chapter 4 (Fieldwork and Response):"In each of previous waves, all interviews were conducted face-to-face CAPI interviews. However, in wave 5, in some cases when the respondent fear of being infected by COVID-19, or when he/she lives in a nursing home where visits are not allowed, the video interview was conducted after it was approved by CHARLS headquarter."

These changes—ranging from content reduction to deviations in interview modality—have implications for data completeness, comparability, and measurement consistency. Therefore, to preserve the integrity and comparability of our analysis across waves, we excluded the 2020 wave and limited our study to pre-pandemic data.

Rationale for Covariate Selection and Exclusion of Other Potential Confounders

The selection of covariates in this study was informed by prior literature and their well-established associations with depressive symptoms among middle-aged and older adults. Specifically, we included gender, age, education level, marital status, residence, drinking frequency, smoking status, and the number of chronic diseases, as these factors have consistently been shown to confound the relationship between sleep and mental health outcomes in epidemiological studies. Other potential confounders—such as baseline physical activity (PA), cognitive function, social engagement, and anxiety symptoms—were also considered during model development, but were ultimately excluded from the final analysis for the following reasons:

High Proportion of Missing Data:

Physical activity data were missing for more than 40% of participants at baseline, which would have substantially reduced the sample size and statistical power, and potentially introduced selection bias if only complete cases were analyzed.

Temporal and Causal Considerations:

Cognitive function, social engagement, and anxiety symptoms are not only potential confounders but may also function as intermediate variables or consequences of depressive symptoms. For example, declines in cognitive function and reduced social engagement may result from, as well as contribute to, the onset of depression. Similarly, anxiety symptoms often co-occur with or follow depressive symptoms. Including such variables in the adjustment set could lead to over-adjustment bias or collider bias, thereby obscuring the true association between sleep duration and depressive symptoms [1,2].

Data Availability and Consistency:

Some variables—such as cognitive function and anxiety symptoms—were not consistently measured or validated across all survey waves in the CHARLS, limiting their utility for longitudinal adjustment.

To ensure methodological rigor and maintain model parsimony, only those covariates with strong theoretical and empirical support, adequate data completeness, and consistent measurement were included in the final models. The exclusion of additional variables was based on best-practice principles for confounder selection in epidemiological research, with reference to recent recommendations

References:

[1] VanderWeele TJ. Principles of confounder selection. Eur J Epidemiol. 2019;34(3):211-219.

[2] Schisterman EF, Cole SR, Platt RW. Overadjustment bias and unnecessary adjustment in epidemiologic studies. Epidemiology. 2009;20(4):488-495.
